# Recent developments in national Aboriginal and Torres Strait Islander health strategy

**DOI:** 10.1186/1743-8462-1-3

**Published:** 2004-11-18

**Authors:** Ian PS Anderson

**Affiliations:** 1 Centre for the Study of Health and Society & VicHealth Koori Health Research and Community Development Unit, Department of Public Health, University of Melbourne, Melbourne, Australia

## Abstract

In this paper I will describe some of the sentinel events in Aboriginal and Torres Strait Islander health policy and strategy during 2003 and the early part of 2004. This will involve discussion on the:

• National Strategic Framework in Aboriginal and Torres Strait Islander Health

• National Strategic Framework for Aboriginal and Torres Strait Islander Peoples Mental Health and Social and Emotional Well Being 2004–2009

• National Aboriginal and Torres Strait Islander Health Performance Framework

• The roll-out of the Primary Health Care Access Program

• The National Aboriginal and Torres Strait Islander Social Survey and the National Indigenous Health Survey

These developments are consistent with a policy agenda that has evolved, in general terms, since the release of the National Aboriginal Health Strategy in 1989. However, I will also consider significant developments in the broader context for Aboriginal and Torres Strait Islander affairs, particularly the decision made in early 2004 by the Howard government to abolish the Aboriginal and Torres Strait Islander Commission (ATSIC). While the key events and developments that are reported in this paper elaborate on an agenda that has been developing for more than a decade, the decision to abolish ATSIC is likely to have a revolutionary impact on the future development of Aboriginal health strategy.

## Introduction

Following the lead of the National Aboriginal Health Strategy (NAHS) [1], national strategy in this field has focussed on health sector reform and the development of inter-sectoral strategies to improve Indigenous health outcomes. In 1995, the health portfolio assumed responsibility for the management of the Australian government's Aboriginal health program from the Aboriginal and Torres Strait Islander Commission (ATSIC). Since that time, mechanisms have been established that provide a platform for collaborative, inter-governmental planning, engaging with both the Aboriginal community sector and the non-health sectors of government [2-4]. The key elements of this national planning framework include:

• Framework Agreements in Aboriginal and Torres Strait Islander Health (multi-party agreements between the Australian government; State and Territory governments; the Aboriginal and Torres Strait Islander Commission and the Aboriginal community controlled health sector);

• Joint Planning Forums (established at a jurisdictional level with responsibility for the developing State and regional Aboriginal and Torres Strait Islander health plans).

The NAHS has been the guiding framework for action in this field since it was endorsed in 1989. Consequently, it was significant that the Australian Health Ministers Conference endorsed its successor on the 31^st ^of July 2003, the "National Strategic Framework for Aboriginal and Torres Strait Islander Health" (hereafter, the "National Framework"). Agreement has also been recently brokered that details strategies for Indigenous social and emotional well-being, which is one of the Key Result Areas for the "National Framework". Significant progress has also been made in the development of a national performance management framework for Aboriginal and Torres Strait Islander health that aligns with the "National Framework".

The agenda in Aboriginal and Torres Strait Islander health strategy that was adopted by the health portfolio post 1995 had focussed on reform priorities focussed on the development of [3]:

• The capacity of primary health services to respond to Aboriginal and Torres Strait Islander health need (with a particular focus on financing and workforce);

• disease and risk strategies that aimed to improve Aboriginal and Torres Strait Islander health outcomes;

• the evidence base for policy and practice in this sector (through strategic research and improvements in the quality of health and related data).

With respect to primary care capacity, the roll-out of the Primary Health Care Access Program (PHCAP) continues to be one of the central planks of this agenda and I will provide an overview of recent progress. Significant progress has also been made over the last couple of years in the development of the Australian Bureau of Statistics Indigenous Survey program, which promises to enhance the information available to assist decision-making within the sector. I will provide a report on the recent developments in the roll-out of this program.

While the recent developments in Aboriginal and Torres Strait Islander health policy and strategy represent an evolution of a health reform agenda that has been developing for more than a decade, the abolition of ATSIC points to a much more revolutionary change in the broader institutional and programmatic context for Aboriginal affairs. ATSIC had play a critical role in integrating Australian government programs in Indigenous affairs and providing an institutional structure that facilitated Aboriginal and Torres Strait Islander input into policy and program development. ATSIC, for instance, continued to play a role in health strategy following the transfer of specific health program responsibilities in 1995. It retained, for instance, responsibility for the delivery of environmental health service. A memorandum of understanding was developed between the Department of Health and Human Services and ATSIC to support collaboration between the sectors [6]. Consequently, the decision to abolish the ATSIC and radically overhaul of the administration of Commonwealth programs in Aboriginal Affairs has potential implications for national health strategy. These are discussed later in this paper.

## Discussion

### National Strategic Framework in Aboriginal and Torres Strait Islander Health

The National Aboriginal and Torres Strait Islander Health Council (NATSIHC) oversaw the development of the "National Framework". However, this process was stalled by political conflict between the key stakeholders. In December 2000, Council members representing the National Aboriginal Community Controlled Health Organisation (NACCHO) resigned in protest over a consultation draft of the 'National Framework'. NACCHO, which is the peak body representing the Aboriginal community controlled health sector, was concerned with [7]:

The way the Draft is written distances Aboriginal and Torres Strait Islander people, undermines the concept of Aboriginal community control of primary health care service delivery and diminished structures which NACCHO believe are still useful. The document's tone and language is wrong in a number of ways...

Following further negotiation, NACCHO withdrew its resignation, and the NATISHC was reconstituted with revised membership and Terms of Reference [8]. Despite this successful outcome, this ruction in the relationship with NACCHO illustrates the tenuous nature of partnership structures and processes in this sector – an issue that I will return when discussing the issues that may potentially flow on following the abolition of ATSIC.

The agreed "National Framework" consists of two documents:

• The "National Strategic Framework for Aboriginal and Torres Strait Islander Health – Framework for action by Governments", which sets out a five- to ten-year reform agenda in 9 key result areas [5].

• The "National Strategic Framework for Aboriginal and Torres Strait Islander Health – Context", which outlines the rationale for the Framework and its context [9].

There are nine Key Result Areas set out in the Framework including [5]:

• Community controlled primary health care: building community capacity so that individuals and communities can better address and manage their own health needs.

• Health system delivery framework: improving the responsiveness of the mainstream health system to Indigenous Australians and developing stronger partnerships between mainstream and Indigenous-specific services.

• A competent health workforce: improving the training, supply, recruitment and retention of appropriately skilled health professionals, health service managers and policy officers in both mainstream and Indigenous-specific health services.

• Emotional and social well-being: improving outcomes with respect to mental health, suicide, family violence, substance misuse and male health (through non-health sectors strategies).

• Environmental health: improving the delivery of safe housing, water, sewerage and waste disposal.

• Wider strategies that impact on health: undertaking action in portfolios outside the health sector and implementing health gain strategies in the areas of education, employment transport, food and nutrition, custodial health, aged and disability services, recreation and exercise.

• Data, research and evidence: aiming to improve the quality of information about how well the health sector is meeting the needs of Indigenous Australians.

• Resources and finance: aiming to provide an optimal level of resources for Indigenous health commensurate with levels of need, costs of delivering services and community capacity to deliver health outcomes.

• Accountability: both to communities and to governments for the delivery and effectiveness of health services.

The "National Framework" was endorsed as a plan to guide all Australian governments in a coordinated, collaborative and multi-sectoral approach to achieving Aboriginal and Torres Strait Islander health gain over the next decade. It does not have a specific funding program attached to its implementation, although arguably, the roll-out of the Primary Health Care Access Program (described later) will provide additional capacity to the implementation of the "National Framework". It is also possible that the National Framework will guide the allocation of any new resources made available through the joint planning processes established under the Framework Agreements.

To further these ends, it is significant that the "National Framework" was endorsed through each government's cabinet process, providing a whole-of-government commitment to its implementation in each State and Territory and at the Commonwealth level. Each jurisdiction is developing its implementation plan against which it will report annually on progress and outcomes in health portfolios and biennially on whole of government progress. The plans will identify the specific strategies and timeframes for each action area. The National Aboriginal and Torres Strait Islander Health Council will develop a plan for an independent mid-term and final evaluation.

### The National Strategic Framework for Aboriginal and Torres Strait Islander Peoples Mental Health and Social and Emotional Well Being 2004–2009

The "Social and Emotional Well Being Framework (SEWB Framework)" [10] is based on Aboriginal health values that emphasise the need for a holistic and 'whole of life' approach to achieving the conditions for well-being. Although this framework encompasses the traditional field of mental health, these issues are situated in an approach that also addresses the emotional and social well-being of Indigenous Australians and their communities.

The nine guiding principles for the "SEWB Framework" were been extracted from "Ways Forward" [11], an earlier strategy that established the importance of this holistic approach in this area of health. In supporting this holistic approach the "SEWB Framework" articulates strategies that support self-determination and culturally valid understandings of health. It further recognises the impact of trauma, grief, loss, discrimination and human rights issues on the social and emotional well being of Aboriginal and Torres Strait Islander communities.

In 2003 the Social Health Reference Group (SHRG) (established to oversee its development) conducted extensive consultations on a draft framework document. Since then the 'SEWB Framework' has endorsed by the NATSIHC and the National Mental Health Working Group in November 2003, and the Standing Committee on Aboriginal and Torres Strait Islander Health in December 2003. It was anticipated that the final 'SEWB Framework' document would be endorsed out of session by the Australian Health Ministers Advisory Council by the middle of 2004.

### The Aboriginal and Torres Strait Islander Health Performance Framework

The development of the Aboriginal and Torres Strait Islander Health Performance Framework has built on the foundations of earlier work which has established the key elements of this framework, including the:

• national performance indicators in Aboriginal and Torres Strait Islander health for the Australian Health Ministers Advisory Council [3];

• service activity reporting for Aboriginal community controlled health services [12];

• Australian government health portfolio indicators [13]; and

• the reporting against key indicators of Aboriginal and Torres Strait Islander disadvantage for the Council of Australian Governments [14].

It is intended that the Aboriginal and Torres Strait Islander Health Performance Framework will both integrate those government performance reporting processes that have already been developed; streamline reporting processes in Indigenous health and, ensure the strategic management of policy relevant and quality information in published reports (such as the National Health Performance Committee, the Productivity Commission's Report of Government Services and the Indigenous Disadvantage Report) [15]. As starting point, the National Health Performance Committee Framework, which has already been endorsed by the Australian Health Ministers Conference will be used as a guide to the relevant measurement domains for Aboriginal and Torres Strait Islander specific framework [15]. It is also intended that the Aboriginal and Torres Strait Islander Health Performance Framework will support the implementation of the 'National Framework' by:

• mapping the relationship between the Key Result areas of the 'National Framework' and key domains of health performance (effectiveness, safety, responsiveness etc); and

• identifying priorities for data development and improvement based on priorities of the 'National Framework'.

### Roll-out of the Primary Health Care Access Program

The Primary Health Care Access Program (PHCAP) was introduced in the 1999–2000 Federal Budget to improve access to primary health care for Aboriginal and Torres Strait Islander people. PHCAP achieves this by funding increased primary health care provision, such as additional general practitioners, nurses, Aboriginal Health Workers, and through preventive and health promotional activities, such as diabetes education and management. Funds are also used for supports to service provision such as capital works and equipment. The program also aims to work with existing health services to ensure they are responsive to the needs of Aboriginal and Torres Strait Islander people.

On average across Australia, PHCAP aims to bring the level of Commonwealth funding for Indigenous primary health care to three times the average MBS usage for all Australians. The key objectives of PHCAP are [3]:

• Increased availability of appropriate primary health care services where they are currently inadequate;

• Local health systems that better meet the needs of Aboriginal and Torres Strait Islander people; and

• Individuals and communities that are empowered to take greater responsibility for their own health.

Services can be provided through a mix of arrangements, including Indigenous specific, mainstream or a combination of these. Funding can also be used to support mechanisms to assist service providers to deliver better services and enable individuals and communities to become more involved in improving their health.

Up until March 2004, new and additional services have been funded in Central Australia (5 regions), Queensland (3 regions) and South Australia (4 regions) through PHCAP, as well as continued funding of services provided through the former Aboriginal Coordinated Care Trials (Yael Cass, Office for Aboriginal and Torres Strait Islander Health, Australian Department of Health and Ageing, personal communication).

During 2003 a more streamlined approach to the management of PHCAP rollout was developed [4,16], resulting in more than 200 proposals to improve access to primary care being developed in each state and territory. This was in discussions with members of the state or territory forum or partnership, and drawing on regional plans and other work that has been undertaken over the last several years.

From these proposals, $11.8 million in funding was approved on 14 March 2004 for:

• additional health professional and support staff, for example, over 20 more health professional positions in the Kimberley region of WA;

• capital works for health clinic upgrades and the construction of staff housing in remote communities;

• minor capital purchases such as medical equipment; and

• one-off health promotional activities and health board support and training.

Longer term strategies around enhancement of local service systems, to ensure they are more accessible for Aboriginal and Torres Strait Islander people, and ensuring the commitment by state/territory governments to at least maintain their funding commitments, will continue to be pursued. While the PHCAP program has provided a significant injection of resources into what is generally considered an inadequately funded primary heath care system, the amount made available through this program still does not meet its programmatic benchmarks and targets [4].

### Roll-out of the Australian Bureau of Statistics Indigenous Survey Program

One of the development priorities established by the heath portfolio when it took responsibility for the administration of the Aboriginal health program was to develop the evidence base to support policy reform and the development of health service capacity [3].

The National Health Survey, undertaken by the Australian Bureau of Statistics with funding support from the Australian Department of Health and Ageing, collects information about the health status, use of health services and facilities, socio-economic status and health-related aspects of the lifestyle of Australians. The Indigenous component of this survey aims to benchmark information on a range of health issues and enable comparisons between the health characteristics of Indigenous and non-Indigenous Australians and to allow trends in the health of Indigenous Australians to be monitored over time.

The Indigenous Health Survey that was run in 2001, collected data from approximately 3,500 individuals which was reportable at the national level [17]. In 2004, the Indigenous Health Survey will collect information from 11,000 Indigenous participants in order to be able to provide statistics at the national and State/Territory levels, and some geographical areas. It will also, for the first time, provide information on mental health. It is anticipated that the data collected will be reported in 2005.

In parallel with the health survey program the Australian Bureau of Statistics collected data for the 2002 National Aboriginal and Torres Strait Islander Social Survey from August 2002 to April 2003 [18]. It is planned to repeat this survey a six yearly intervals. A summary of findings has been published that covers topics such as family and culture, health, education work, income and housing law and just and transport.

### A revolution in program administration in Aboriginal Affairs

On 20 April 2004, the Prime Minister, John Howard and the Minister for Aboriginal Affairs, Senator Amanda Vanstone, announced the intention of the Australian government to abolish the ATSIC.

ATSIC had been established in 1989 when the program responsibilities of the Commonwealth Department of Aboriginal Affairs and the Aboriginal Development Corporation were merged into a structure that enable the regional allocation of resources through elected regional councils. The board of Commissioners, elected by ATSIC regional councils, was responsible for national policy development and the oversight of national programs. At the Commonwealth level, ATSIC had the lead agency responsible for the administration of a range of programs such as: community development and employment (CDEP); housing and infrastructure; cultural heritage, broadcasting services; legal services; native title, land rights and the Indigenous land fund, etc.

The agency also played a critical role in co-ordinating and integrating the Aboriginal strategy across the different government program areas. ATSIC and the health portfolio had collaborated in the implementation of health infrastructure priority projects [19]. The Memorandum of Understanding developed between the two portfolios enabled collaborative planning across a range of programs that impacted on Indigenous health outcomes. The advantage of this institutional arrangement for cross sectoral strategy is that these programs might otherwise have been dispersed across a number of different government departments or instrumentalities. Further, ATSIC provide a structure for engaging Indigenous participation that broader than the sector specific mechanisms.

ATSIC played a pivotal institutional role in the development of 'whole of government' strategies across the Australian government. It was in effect the only institutional mechanism (with the exception of time limited inter-departmental committees) that enabled this. This was until the Council of Australian Governments (COAG) resolved (in 2000 and 2002) to trial, in up to 10 regions across the country, innovative administrative arrangements, developed in partnership with Indigenous communities, which aimed to provide "more flexible programs and services based on priorities agreed with communities" [20].

From its first term in 1996, the Howard Coalition government had a conflictual relationship with the Commission. However, government confidence in the ATSIC Board deteriorated significantly under the chairmanship of Geoff Clark (first elected chairperson in 1999) to the extent that the Minister for Indigenous Affairs suspended him on the ground of misbehaviour (under section 40 of the ATSIC Act 1989) [21]. A review of ATSIC was undertaken during the period December 2002–October 2003. It recommended that ATSIC should be retained as the primary vehicle for representing the aspirations of Aboriginal people to all levels of government and that its existing program responsibilities should also be retained pending a determination of its role in the context of [a] broader examination of service delivery [22]. The review also recommended a comprehensive program of reform primarily focussed at strengthening the capacity of regional councils and improving the relationships between ATSIC and the Australian government and between ATSIC's elected and administrative arms. Prior to the completion of the review the Coalition government moved to structurally separate ATSIC into an elected arm (ATSIC) and an executive agency, Aboriginal and Torres Strait Islander Services (ATSIS). ATSIS retained, under Ministerial delegation, program administrative responsibilities.

The Federal cabinet, nevertheless, resolved to a more radical agenda than outlined in the review findings, and announced its intention to abolish ATSIC, its regional councils and the mainstreaming of the administration of all the programs for which ATSIC had been responsible. It is proposed that the elected advisory structures will be replaced by a government appointed national council. It is also proposed that Indigenous specific program dollars will be quarantined and a whole of government approach is to be developed for the delivery of Indigenous specific funding. The key elements of this reform package have been positioned within the broader context of a government commitment to reforms aimed at producing "'joined up' government and the 'seamless' delivery of programmes" [23]. This new framework for Indigenous policy and program administration also include the establishment of a:

• Ministerial Taskforce: which would operate as a cabinet committee, provide collaborative leadership at a government level and set strategic directions.

• Secretaries group: which would support Ministerial decision-making, coordinate across government agencies, and oversight annual reporting.

• National Indigenous Council: in which the Minister would appointment Indigenous leaders in health, education, employment, law and justice to provide advice and monitor performance.

The proposed mechanisms and structures that would be established to deliver this 'joined-up' framework including regional partnership agreements and community shared responsibility agreements (detailing mutual obligations). It is also proposed to establish Indigenous co-ordination centres which will provide a single shopfront in regional and remote Australia for indigenous specific programs, lead the negotiation of regional partnerships an shared responsibility agreements but maintain line responsibility to mainstream departments.

The impact of this radical reform agenda to national Aboriginal health strategy is difficult to predict. One the one hand the actual changes to the administration of the health program is insignificant (leaving aside some potentially critical issues in budget development). A mainstream department has administered this program since 1995. One the other hand, the implementation of this reform agenda could have potentially very significant consequences for the development of inter-sectoral strategies in Indigenous health. This depends on the success in implementing the new mechanisms, and on their effectiveness. Furthermore, the political consequences of this radical agenda on the relationship between the Australian government and Aboriginal and Torres Strait Islander peoples have yet to really become clear. Specific partnership arrangements that have been developed within the health sector are tenuous – as is evident by the politics in the development of the "National Framework". These partnerships are critical to successful implementation of strategy in Indigenous health. Consequently, deterioration in the broader relationship between Indigenous Australians and the Australian government may have significant negative consequences for the partnership processes specific to the health sector. Even though 2003 was a year in which policy and strategy in Indigenous health made no or new radical departures, it was a year of considerable tumult in relations between the Australian government and Indigenous peoples. The ramifications of this are only now beginning to unfold.

## Abbreviations

ATSIC Aboriginal and Torres Strait Islander Commission

NAHS National Aboriginal Health Strategy

NATSICH National Aboriginal and Torres Strait Islander Health Council

NACCHO National Aboriginal Community Controlled Health Organisation

PHCAP Primary Health Care Access Program

## Competing interests

The authors declare that they have no competing interests.
